# Effects of empagliflozin on gonadal functions of hyperglycemic male wistar rats

**DOI:** 10.1371/journal.pone.0305636

**Published:** 2024-06-17

**Authors:** Roba Bdeir, Nour A. Al-Sawalha, O’la Al-Fawares, Lama Hamadeneh, Alia Khawaldeh

**Affiliations:** 1 Department of Allied Health Sciences, Faculty of Nursing, Al-Balqa Applied University, Al-Salt, Jordan; 2 Department of Clinical Pharmacy, Faculty of Pharmacy, Jordan University of Science and Technology, Irbid, Jordan; 3 Department of Medical Laboratory Sciences, Faculty of Science, Al-Balqa Applied University, Al-Salt, Jordan; 4 Department of Basic Medical Sciences, Faculty of Medicine, Al-Balqa Applied University, Al-Salt, Jordan; 5 Faculty of Pharmacy, AL-Zaytoonah University of Jordan, Amman, Jordan; 6 Department of Medical Laboratory Sciences, Faculty of Allied Medical Sciences, Jadara University, Irbid, Jordan; Zagazig University, EGYPT

## Abstract

Empagliflozin (EMPA) showed antiapoptotic, oxidative and anti-inflammatory potential effect. EMPA attenuates the inflammation and oxidative stress biomarkers in patients with heart failure while significantly decreases the malondialdehyde (a lipid peroxidation marker) levels in the plasma of diabetic patients. The present study examined the effects of moderate hyperglycemia on reproductive function. Sixty male Wister rats ‎ were divided and randomly allocated into four groups of 15 animals each‎‎. Diabetes was induced by a single intraperitoneal injection of a prepared solution containing STZ diluted in 0.1 M sodium citrate buffer (pH 4.5) at a dosage of 40 mg/kg body weight in selected in groups II and III for seven days before starting the treatment with EMPA. The current study revealed that EMPA for eight weeks prevented testicular high glucose-induced oxidative stress markers such as penile nitric oxide (NO), glutathione peroxidase (GPX) and total anti-oxidant capacity (TAC) in STZ-induced hyperglycemia in a rat model. In addition, EMPA ameliorated the high levels of endogenous Interleukin-6 (IL-6) present in gonads in response to an acute inflammatory found in the hyperglycemic STZ-induced rats. The present study further suggested the protective effects of EMPA and how it has a beneficial role and can effectively attenuate hyperglycemia-induced testicular oxidative damage and inflammatory markers as well as androgen dependent testicular enzymes activity as a protective role against the consequences of hyperglycemia and male sub-infertility.

## Introduction

Diabetes mellitus (DM) is a lifelong metabolic disease with inappropriately elevated blood glucose levels (blood glucose > 126 mg/dL) [1]. Several serious complications such as cardiovascular diseases, renal disease, neuropathy, retinopathy, lower-extremity amputation, skin and digestive problems as well as sexual dysfunction [2–4]. It is worth mentioning that DM effected 9.3% of global population in 2019 and the percentage is presumed to rise to 10.2% by 2045 [5].

EMPA‎ is an anti-diabetic medication that particularly inhibits sodium glucose cotransporter-2 (SGLT2) [6]. The mechanisms behinds considering it as a selective SGLT 2 inhibitor is the ability to lower fasting and postprandial plasma glucose by boosting 24-hour urine glucose excretion in a non-insulin-dependent manner [7]. It has been demonstrated that EMPA has a minimal chance of inducing hypoglycemia comparing to other antidiabetic drugs as it has insulin-independent mechanism of action [8]. Current studies revealed that EMPA might have powerful antiapoptotic, antioxidant, and anti-inflammatory effects as well [9]. Indeed, the administration of EMPA induced significant increase in sex hormones, attenuated the oxidative stress, and improved the inflammatory processes in rats with cadmium induced testicular toxicity [10–12]. These properties may give a role to EMPA in amelioration of diabetes-induced reproductive complications.

As mentioned above, insulin deficiency can damage the hypothalamus, pituitary gland, gonads, and perigonads [13]. This can result in decreased sex hormone secretion, including gonadotropin-releasing hormone (GnRH), follicle stimulating hormone (FSH), luteinizing hormone (LH), and testosterone, as well as structural harm to the male reproductive system, including testicular atrophy, stromal cell atrophy, damage to the seminiferous tubules, damage to spermatogenic cells, and other injuries [14, 15]. These side effects has an impact on male fertility and reproductive health [16]. Various studies over the years indicate AMP-activated protein kinase (AMPK) is involved in the regulation of reproductive function [17]. Briefly, AMPK serves as a major energy sensor to monitor the supply of nutrients, affect the secretion of GnRH and gonadotropins, regulate gonadal steroid hormone production and link energy status with fertility through the hypothalamus and pituitary [18, 19]. Moreover, AMPK itself is also expressed in the gonad, and is involved in regulating spermatogenesis [17, 20].

Steroidogenic acute regulatory protein (StAR), a mitochondrial cholesterol delivery protein, plays a protective role in systemic inflammation and insulin resistance in obese mice [21]. However, it is well known that StAR gene up-regulates the expression of reproductive genes [22]. Oxidative stress has been linked to male infertility, as it is a prevalent pathology alongside reproductive issues [23]. In men with diabetes or obesity, elevated reactive oxygen species (ROS) levels may serve as prognostic indicators in subfertile patients [24]. An investigation showed that oxidative stress in the heart of diabetic rats has been mitigated by using EMPA [25]. In a clinical trial carried out in 2017, Larsen et.al. reported that the administration of EMPA significantly decreases the malondialdehyde (a lipid peroxidation marker) levels in the plasma of diabetic patients [26]. Furthermore, EMPA attenuates the inflammation and oxidative stress biomarkers in patients with heart failure [27].

Given the crucial evidence of the importance of steroidogenesis in reproduction and the rate-limiting step of the StAR protein, we aim to investigate the sex hormonal levels as well as the gene expression of the StAR protein. The present study examined the effects of moderate hyperglycemia on reproductive function. Thus, 40 mg/kg dose of intraperitoneal Streptozotocin (STZ) is the most used diabetogenic chemical for inducing hyperglycemia in Wistar rat (*Rattus norvegicus*) models [25]. Various biochemical assay of testicular oxidative stress parameters and testicular enzyme activities, conducting histopathological examination under light microscope and finally studying the gene expression to better understand the molecular mechanisms of key regulatory enzymes and their effect on reproduction and gonad functionality.

## Materials and methods

### Animals and ethical approval

Sixty male Wister rats (9–10 weeks old) weighing around 180–200 g were housed in compliance with the Animal Care and Use committee’s guidelines at Jordan University of Science and Technology, approval number: 20210433. Rats were maintained on a twelve-hour light/dark cycle at room temperature with unrestricted access to water and food and were given an acclimation period of 3 days (72 hours) prior to use start of the experiment.

### Treatments

STZ induced hyperglycemia is the model used to evaluate the activity of hypoglycemic agents. A single intraperitoneal injection of a prepared solution containing STZ dissolved in 0.1 M sodium citrate buffer (pH 4.5) at a dose of 40 mg/kg body weight was administered to induce diabetes in rats in two out of the four groups seven days before starting the treatment with EMPA [25]. Followed STZ injection, the drinking water supplemented with sucrose (15 g/L) was offered for 48 hours to reduce early mortality and overcome the drug-induced hypoglycemia [24]. The fasting plasma glucose was measured one day after injection of STZ. Diabetic rats were included in the study if fasting blood glucose level is >150 mg/dl [26]. The fasting blood glucose was measured every other day by a glucose analyzer (Accu-chek Guide Blood Glucose Meter). The treatment started on day 8 after STZ-injection with EMPA administered by oral gavage at a dose of 10 mg/kg of EMPA and dissolved in 5% hydroxymethylcellulose. The administration is continued for 8 weeks, with the reference of the effective dose of EMPA by Yesilyurt et al. 2021 [28].

### Experimental set up and gonads dissection

The animals were divided and randomly allocated into four groups of 15 animals each. The groups were non-diabetic (control), non-diabetic with EMPA treatment (EMPA), diabetic (STZ) and diabetic with EMPA treatment (STZ + EMPA). After 8 weeks of treatment, the rats were sacrificed by cervical decapitation, a physical euthanasia of small animals by applying pressure to the neck and dislocating the spinal column from the skull or brain [29], and the gonads was dissected. According to Spitz et al. [30], the tissue homogenate was prepared and used for the testicular antioxidant and enzyme activity. All tissue underwent a saline wash to eliminate excess connective tissues and red blood cells. Subsequently, the tissue was finely chopped in 0.05 M phosphate buffer solution with a pH of 7.8, using scissors. The minced tissue was then subjected to homogenization using a Tekmar SDT ultraspeed tissue grinder (with six bursts of five seconds each). All procedures were conducted under ice.

### Blood collection and hormonal levels in serum

After sacrifice the rats by decapitation, blood was collected and serum was separated by allowing the blood to clot at room temperature under dark condition for 1 hour, then centrifuged at 3800 rpm for 15 minutes. Serum was kept at -80°C till analysis. Serum levels of luteinizing hormone, and follicle stimulating hormone, estradiol and testosterone hormones was calculated by enzyme immunoassay method.

### Testicular antioxidant activity

Tissue lipid peroxidation was estimated using thiobarbituric acid reactive substances (TBARS) (Caymanchem, USA, cat. # 10009055). The activity of superoxide dismutase (SOD) (Sigma- Aldrich Crop., USA, cat. # 19160), catalase (Caymanchem, USA, cat. # 707002), GPX (Sigma- Aldrich Crop., USA, cat. # MBS2516156), NO (R&D systems, USA) as well as level of total antioxidant capacity (TAC) (Caymanchem, USA, cat. # 709001) in testis of rats were measured according to the instructions of the kit’s manufacturer. Furthermore, tissue interleukin 6 (IL-6) (MyBiosource, USA, car. # MBS2701082), transforming growth factor beta 1 (TGF-β1) (R&D systems, USA, cat. # 7754-BH) and total protein present in the tissue homogenate was estimated to evaluate inflammatory response.

### Testicular hormonal levels

The levels of estradiol (cat. # MBS7606334), testosterone (cat. # MBS766199), LH (cat. # MBS2509833) and FSH (cat. # MBS2700328) in the tissue were measured using an ELISA kit according to the manufacturer’s instructions (MyBiosource, USA). Epoch Biotek microplate reader (BioTek) was used to measure the absorbance. The measured levels were normalized to the protein concentration in each sample.

### Assay of testicular enzymes

Acid phosphatase (ACP) (Abcam, USA, cat. # ab83367), glucose-6-phosphate dehydrogenase (G6PD) (MyBiosource, USA, cat. # MBS2702342), sorbitol dehydrogenase (SDH) (MyBiosource, USA, cat. # MBS166115) and Seminal Creatine Kinase (MyBiosource, USA, cat. # MBS1600481) activities in the rats’ testis tissue homogenate was measured according to the instructions of the kit’s manufacturer.

### Histopathological studies

Gonads from three different rats in each group were collected after their euthanasia using cervical dislocation and washed with PBS three times to remove excess blood. Then, tissues were fixed in 10% formaldehyde for 48 hours. Solid sections of the fixed tissues were prepared, dehydrated, cleared infiltrated with paraffin wax and embedded in paraffin blocks. A 3–5 μm thin sections were cut using microtome deparaffinized with xylene, stained with hematoxylin and eosin, and mounted with DPX, two different sections from each duplicated in each group were taken for light microscopic examination and photomicrographs were taken [31].

### Quantitative analysis of *AMPK* and *StAR* gene expression

Total mRNA extraction from testicular tissues was accomplished using the RNeasy Mini Kit (Qiagen, Germantown, MD, USA) following the kit’s procedure. Total RNA collected were used to produce cDNA using SuperScript® VILO™ cDNA Synthesis Kit (Life Technologies, Grand Island, NY, USA). Quantitative real time PCR was used to determine the fold change of *StAR* (Gene ID: 25557) and *AMPK* (Gene ID: 78975) genes normalized to GAPDH (ID: 24383) housekeeping gene using the following primers:

StAR F 5’ GCA AAA GGC CTT GGG CAT AC 3’

StAR R 5’ TCT GTC CAT GGG CTG GTC TA 3’

AMPK F 5’ GCG TGT GAA GAT CGG ACA CTA 3’

AMPK R 5’ GGC CTG TCA ATT GAT GTT CTC C 3’

GAPDH F 5’ CCC CCA ATG TAT CCG TTG TG 3’

GAPDH R 5’ TAG CCC AGG ATG CCC TTT AGT 3’

Quantitative real-time PCR was performed using CFX96 (BioRad, USA) and SYBR® Green Master Mix (BioRad, USA) to a final volume of 20 μL for each reaction mixture. All samples were run in triplicate. The relative gene expression was calculated by using the 2^−ΔΔCT^ method based on the threshold cycles (Ct) values.

### Statistical analysis

One-way analysis of variance (ANOVA) followed by Tukey’s multicomparison test for multiple group statistical analysis will be performed using GraphPad Prism 5® software. *p* values of less than 0.05 were considered to indicate statistical significance. All the results were expressed as mean ± SEM for ten animals in each group.

## Results

### Empagliflozin reduces high glucose-induced oxidative stress

**Fig 1:**
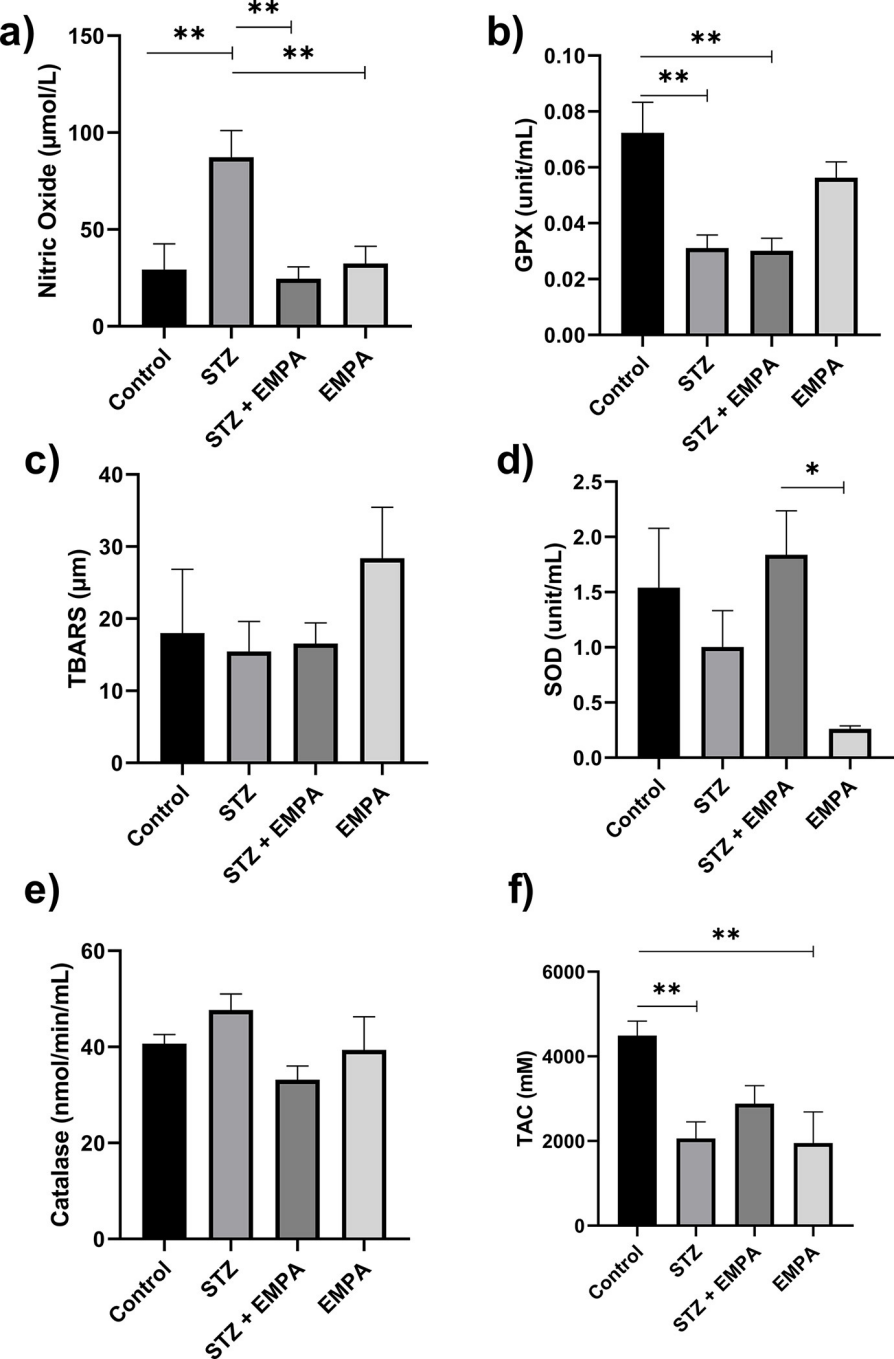
Effects of EMPAgliflozen on the activities of testicular antioxident enzymes in gonads of hyperglycemic rats. Levels of the following antioxidant enzyme activity are represented a) nitric oxide (NO) in μmol/L, b) glutathione peroxidase (GPX) in unit/mL, moles of glutathione oxidized per min per mg of protein, c) thiobarbituric acid reactive substances (TBARS) in μm, d) superoxide dismutase (SOD) in unit/mL, 50% of inhibition of epinephrine auto oxidation per min, e) Catalase in nmol/min/mL, moles of hydrogen peroxide decomposed per min per mg of protein and f) total antioxident capacity (TAC) in mM. Values are given as mean ± SEM, for ten rats per group. Values are statistically significant at * *p* <0.05; ** *p* <0.005.

Multiple comparisons revealed significant results when comparing control and STZ showing that hyperglycermia affected the NO, GPX and TAC levels and and are amongst the high glucose-induced oxidative stress markers to be stimulated by STZ induced diabetes in this model, represented in Fig 1.

The adminstration of STZ significantly increased the NO level compared to control group (87.25 ± 13.83μM/L in STZ group versus 29.33 ± 13.20μM/L in control group, *p* <0.05) (Fig 1A) while it significantly reduced the activity of GPx (0.03 ± 0.004 units/ml in STZ group versus 0.07 ± 10.01 units/L in control group, *p* <0.05) and levels of TAC as compared to control group (2062.00 ± 390.20 μM/L in STZ group versus 4492 ± 340.3 μM/L in control group, *p* <0.05) (Fig 1B and 1F).

Moreover, the level of NO was significantly reduced in STZ + EMPA treated rats compared to STZ treated rats (24.51 ± 6.14μM/L in STZ + EMPA group versus 87.25 ± 13.83μM/L in STZ group, *p* <0.05). However, the activity of GPx (0.03 ± 0.004 units/ml in STZ + EMPA group versus 0.03±0.004 units/L in STZ group, *p* >0.05) and the levels of TAC (2883.00 ± 426.50 mM in STZ + EMPA group versus 2062.00 ± 390.20 mM in STZ group, *p* >0.05) were not altered in STZ + EMPA treated rats compared to STZ treated rats (Fig 1B and 1F).

However, the level of TBARS (18.01±8.83 μM, 15.44 ± 4.16 μM, 16.54 ± 2.87 μM, 28.38 ± 7.07 μM in control, STZ, STZ + EMPA and EMPA groups respectively, *p* >0.05) and the activities of SOD (1.54 ± 0.54 units/ml, 1.003 ± 0.33 units/ml, 1.84 ± 0.33 units/ml, 0.26 ± 0.03 units/ml in control, STZ, STZ + EMPA and EMPA groups respectively, *p* >0.05) and catalase (40.69 ± 1.89 nmol/min/ml, 47.69 ± 3.33 nmol/min/ml, 33.15 ± 2.87 nmol/min/ml, 39.39 ± 6.89 nmol/min/ml in control, STZ, STZ + EMPA and EMPA groups respectively, *p* >0.05) were not affected in any treated groups (Fig 1C–1E).

**Fig 2:**
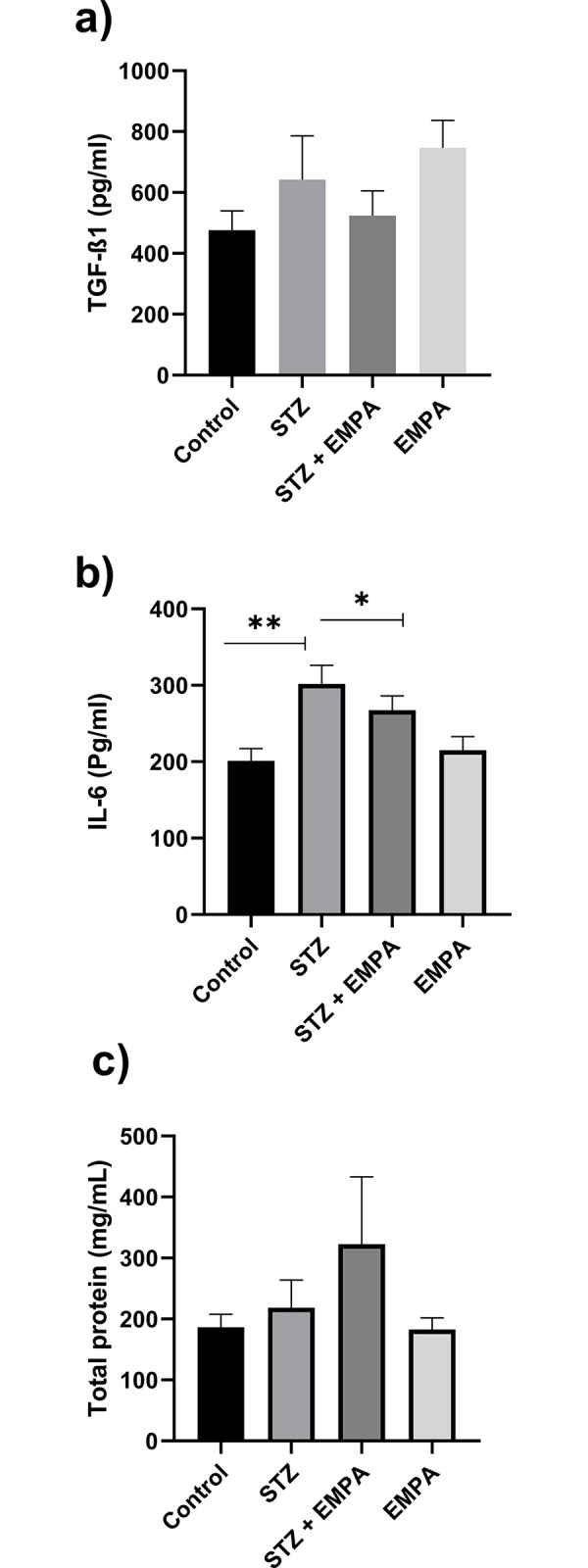
Effects of EMPAgliflozen on the levels of testicular interleukin 6 (il-6), transforming growth factor beta 1 (tgf-β1) and total protein in gonads of hyperglycemic rats. Levels are represented a) Transforming Growth Factor beta 1 (TGF-β1) in pg/ml, b) Testicular Interleukin 6 (IL-6) in pg/ml and c) Total protein in mg/mL. Values are given as mean ± SEM, for ten rats per group. Values are statistically significant at * *p* <0.05; ** *p* <0.005.

The levels of inflammatory biomarkers were examined in rats treated with STZ and EMPA and are represented in Fig 2.

Results showed that STZ significantly increased the level of IL-6, a sign for a crucial antiinflammatory in response to an acute inflammatory responses, compared to control rats (301.90 ± 24.32 Pg/ml in STZ group versus 201.10 ± 15.98 Pg/ml in control group, *p* <0.05) while STZ+EMPA significantly reduced the elevated levels (301.90±24.32 Pg/ml in STZ group versus 267.60 ± 18.62 Pg/ml in STZ + EMPA group, *p* <0.05) (Fig 2B).

However, the levels of TGF-β1 (475.80 ± 63.60 Pg/ml, 642.50 ± 143.5 Pg/ml, 523.6 ± 82.24 Pg/ml, 745.90 ± 91.52 Pg/ml in control, STZ, STZ + EMPA and EMPA groups respectively, *p* >0.05) and total protein (186.40 ± 21.35 mg/ml, 218.5 ± 45.58 mg/ml, 322.50 ± 110.60 mg/ml, 182.70 ± 19.38 mg/ml in control, STZ, STZ + EMPA and EMPA groups respectively, *p* >0.05) were not affected in any treatment group (Fig 2A and 2C).

### The effect of empagliflozin on testicular enzymes

**Fig 3:**
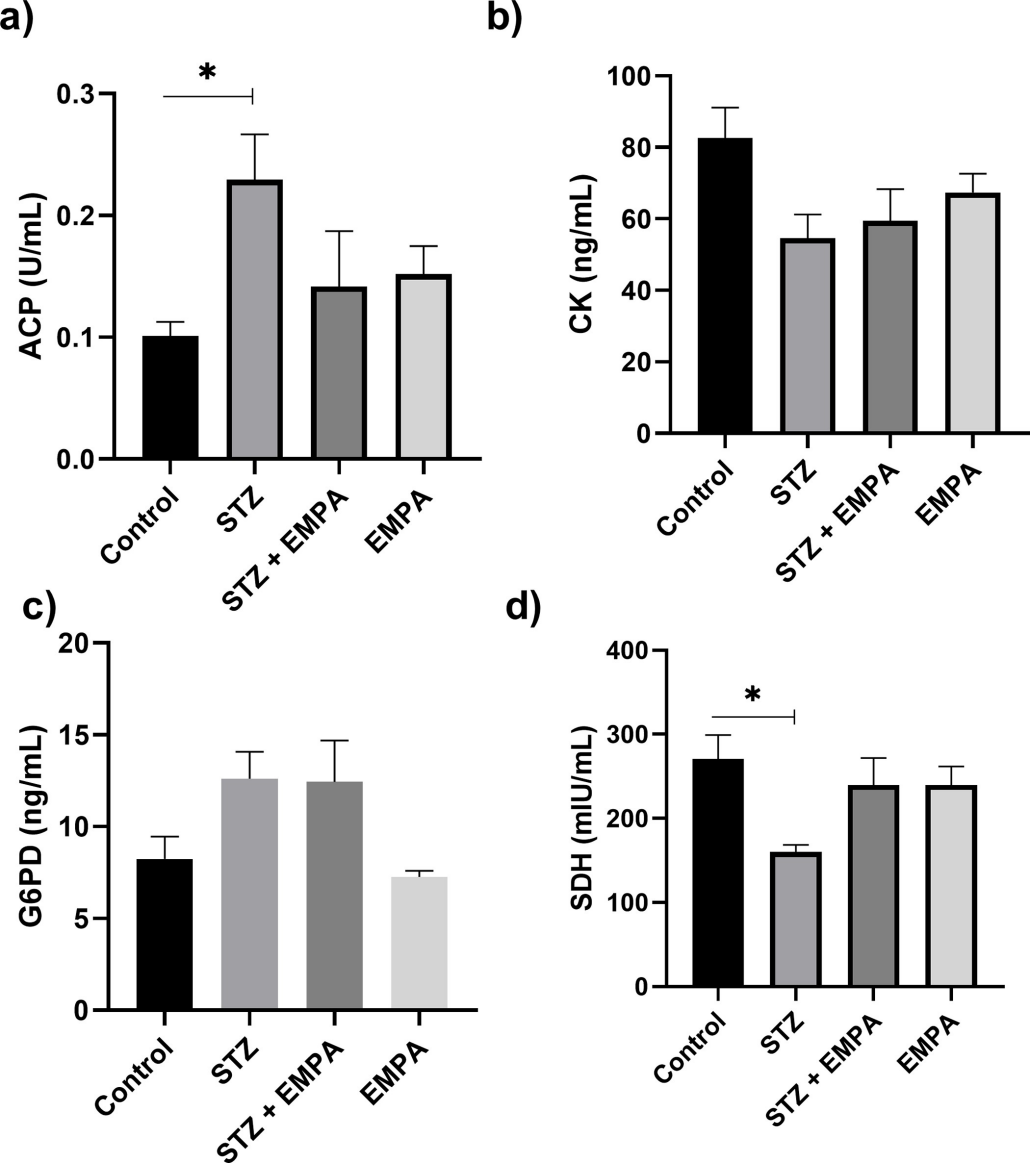
Effects of EMPAgliflozen on the activities of acid phosphatase, glucose-6-phosphate dehydrogenase, sorbitol dehydrogenase and creatine kinase in gonads of hyperglycemic rats. Levels of the following enzyme activity are represented a) Acid Phosphatase (ACP) in unit/mL, b) Creatine Kinase (CK) in ng/mL, c) Glucose-6-Phosphate Dehydrogenase (G6PD) in ng/mL and d) Sorbitol Dehydrogenase (SDH) in mIU/mL. Values are given as mean ± SEM, for ten rats per group. Values are statistically significant at * *p* <0.05.

The levels of testicular enzymes were assessed and Fig 3 revealed that the activity of ACP was significantly increased in STZ treated rats compared to control group (0.23 ± 0.03 U/ml in STZ group versus 0.14 ± 0.03 U/ml in control group, *p* <0.05) (Fig 3A). Moreover, the SDH activity is significantly reduced in STZ group compared to control group (160.10 ± 8.46 mU/ml in STZ group versus 270.40 ± 28.61 mU/ml in control group, *p* <0.05) (Fig 3D). There was a trend of reduced CK level by STZ treatment, but it did not reach a significant level (54.64 ± 6.56 ng/ml in STZ group versus 82.67 ± 8.50 ng/ml in control group, *p* = 0.06) (Fig 3B). However, the G6PD enzyme was not affected by any treatment (8.24 ± 1.21 ng/ml, 12.61 ± 1.46 ng/ml, 12.45 ± 2.25 ng/ml, 7.25 ± 0.34 ng/ml in control, STZ, STZ + EMPA and EMPA groups respectively, *p* >0.05) (Fig 3C).

### The effect of empagliflozin on reproductive hormones

**Fig 4:**
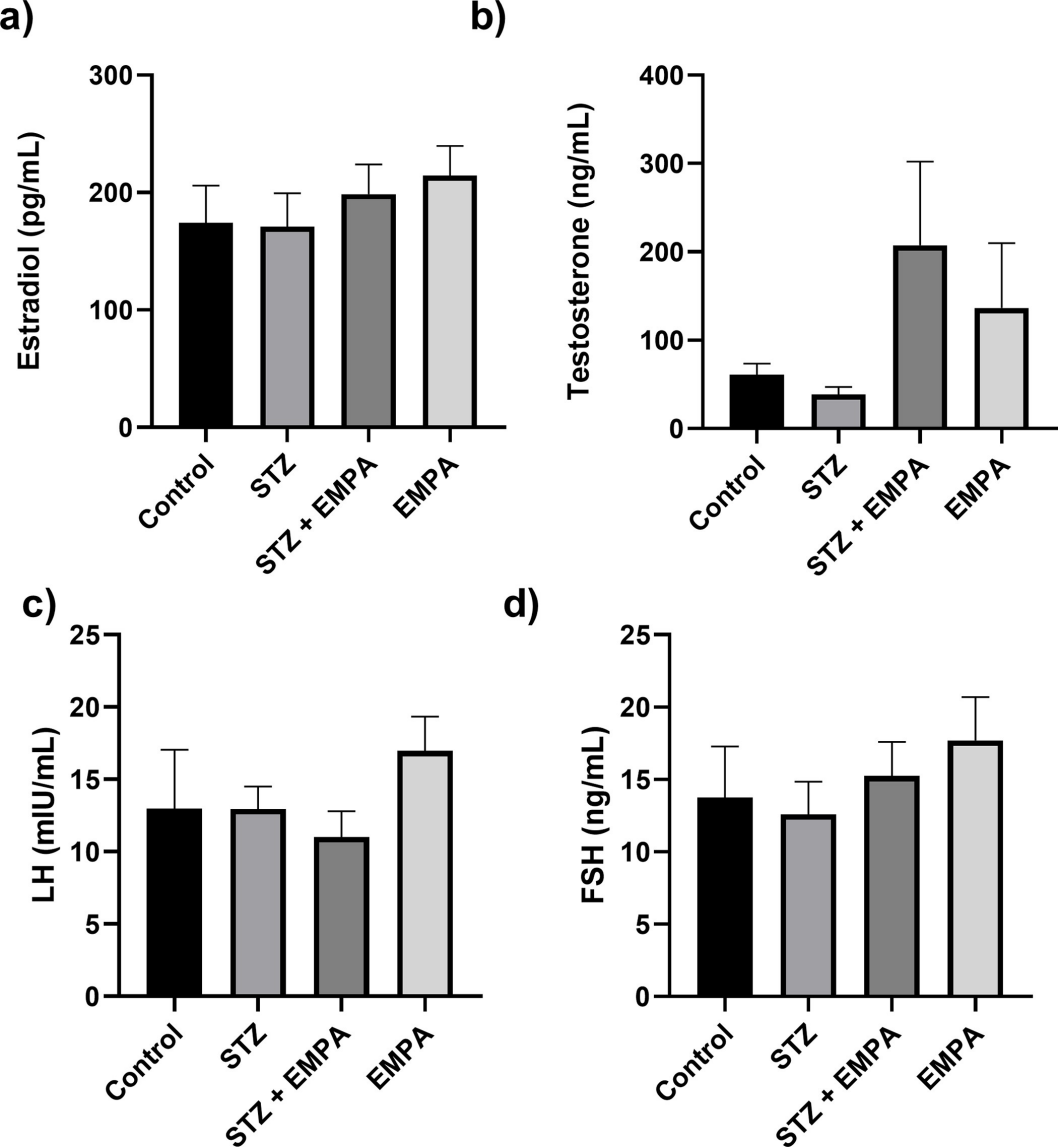
Effects of EMPAgliflozen on the hormonal levels of estradiol, testosterone, luteinizing hormone, and follicle stimulating hormone in gonads of hyperglycemic rats. Levels of the following homones are represented a) estradiol in pg/mL, b) testosterone in ng/mL, c) Luteinizing Hormone (LH) in mIU/mL and d) Follicle Stimulating Hormone (FSH) in ng/mL. Values are given as mean ± SEM, for ten rats per group. Values are statistically significant at * *p* <0.05.

The measured reproductive hormones presented in Fig 4, estradiol (174.10 ± 31.70 Pg/ml, 170.90 ± 28.47 Pg/ml, 198.70 ± 25.30 Pg/ml, 214.30 ± 25.17 Pg/ml in control, STZ, STZ + EMPA and EMPA groups respectively, *p* >0.05), testesterone (61.21 ± 12.34 ng/ml, 38.43 ± 8.73 ng/ml, 207.50 ± 94.82 ng/ml, 136.6 ± 73.20 ng/ml in control, STZ, STZ + EMPA and EMPA groups respectively, *p* >0.05), LH (12.97 ± 4.08 mIU/ml, 12.95 ± 1.56 mIU/ml, 11.00 ± 1.80 mIU/ml, 16.97 ± 2.38 mIU/ml in control, STZ, STZ + EMPA and EMPA groups respectively, *P* value >0.05), and FSH (13.77 ± 3.51 ng/ml, 12.60 ± 2.25 ng/ml, 15.25 ± 2.34 ng/ml, 17.70 ± 3.00 ng/ml in control, STZ, STZ + EMPA and EMPA groups respectively, *p* >0.05), were not affected by STZ or EMPA (Fig 4A–4D). However, it is worth mentioning a trend in an overall increase of testesterone levels in both groups treated with EMPA.

### Histological sections of the rat testicular tissues

**Fig 5:**
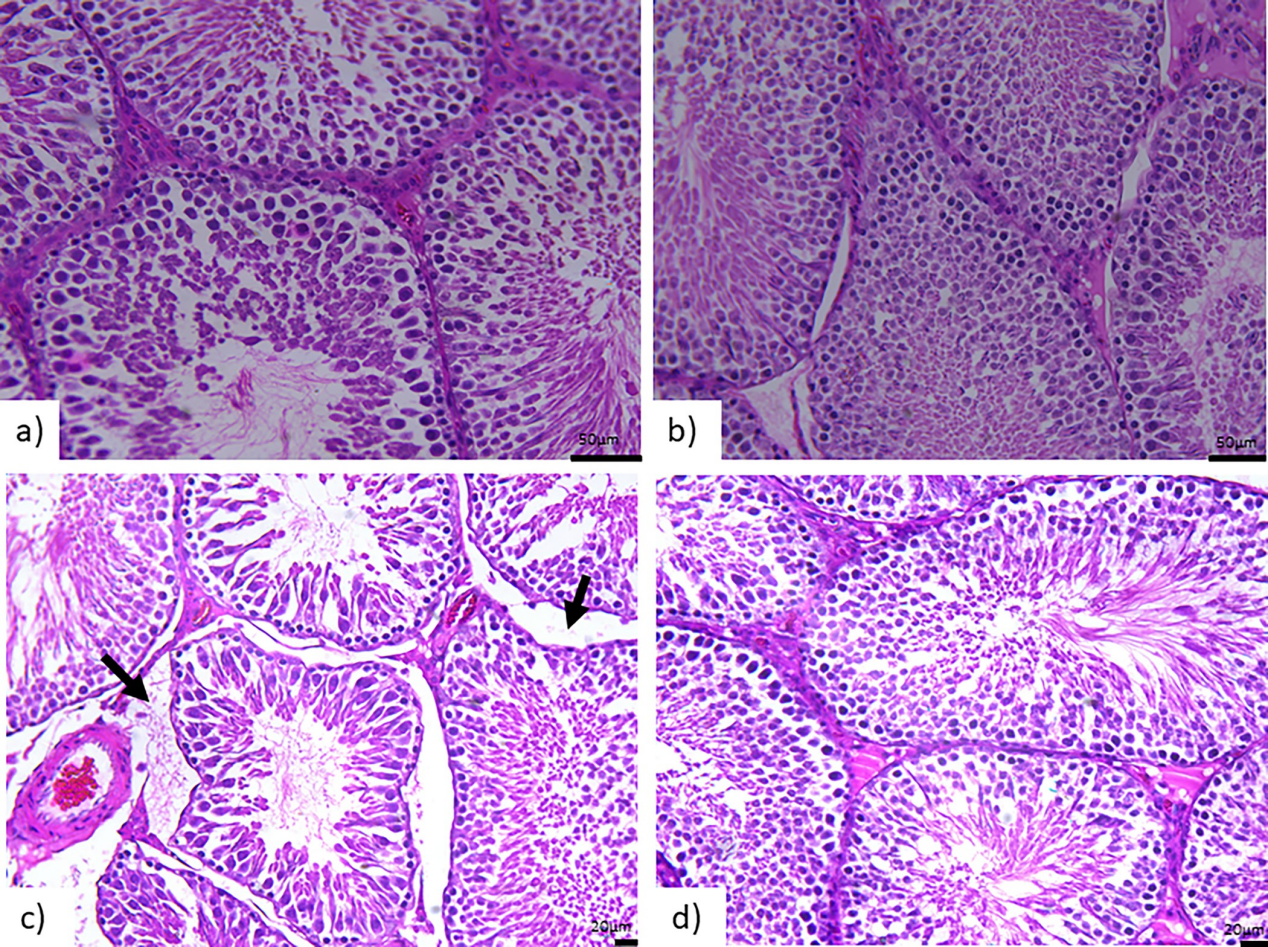
Histological sections of the hematoxylin- and eosin-stained rat testicular tissue sections and the effects of EMPAgliflozen in gonads of hypeglycemic rats. The gonad sections show spermatocytes, spermatogonia, spermatogenic cells and interstitial tissue display Leydig cells and blood vessels. Groups are represented as a) non-diabetic (control), b) non-diabetic with EMPA treatment (EMPA), c) untreated diabetic (STZ) and d) diabetic with EMPA treatment (STZ + EMPA).

In Fig 5, the untreated STZ induced hyperglycemic rats hisotlogical sections illustrate vacuolization of the seminiferous epithelium, congestion of blood vessels, degeneration of spermatogenic cells, and erosion of testicular interstitial cells with the irregular membrane of the seminiferous tube. However, testicular tissues treated with EMPA demonstrate a reduction in damage with normal seminiferous tubules arranged normally.

### The effect of empagliflozin on the relative gene expression of *AMPK* and *StAR*

**Fig 6:**
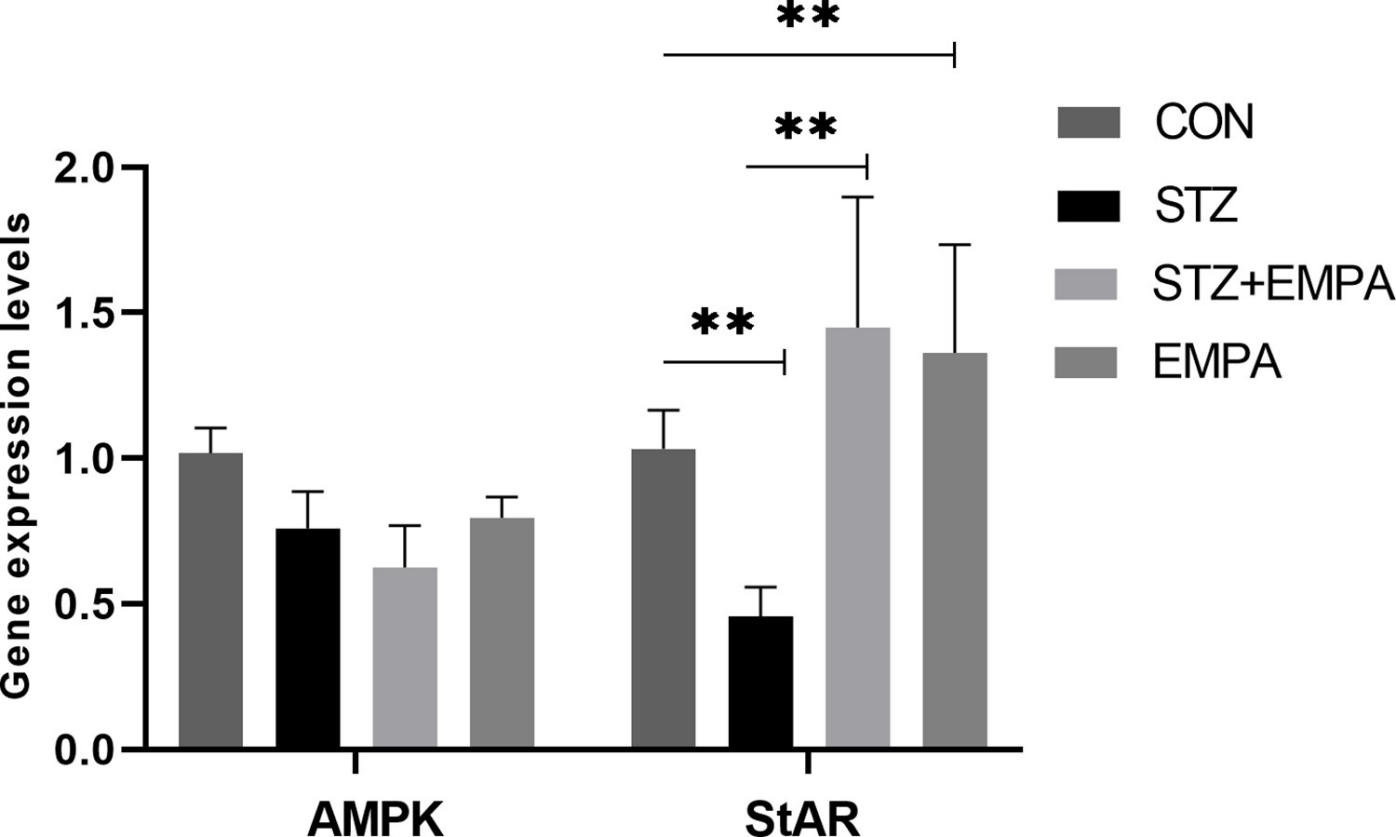
Relative gene expression of *AMPK* and *StAR*. Two folds change in gene expression of *AMPK* and *StAR* genes relative to control undiabetic rat. Values are given as mean ± SEM, for six rats per group. Values are statistically significant at ** *p* <0.005.

Gene expression results presented in Fig 6 showed that neither induction of diabetes nor the use of EMPA has significantly affected the expression of *AMPK* (1.02 ± 0.09 in control group, 0.76 ± 0.13 in STZ group and 0.63 ± 0.14 fold in STZ+EMPA group, *p* >0.05). On the other hand, the expression of *StAR* gene was significantly downregulated in the diabetic group in comparison with the control undiabetic group. Treatment with EMPA significantly increased the expression of *StAR* in diabetic induced rats and control rats when compared with the expression in diabetic group rats.

## Discussion

The current study revealed that EMPA for eight weeks prevented testicular high glucose-induced oxidative stress markers such as NO, GPX and TAC in STZ-induced hyperglycemia in a rat model. In addition, EMPA also ameliorated the high levels of endogenous IL-6 present in gonads in response to an acute inflammatory found in the hyperglycemic STZ-induced rats.

Hyperglycemia increases ROS generation while decreasing antioxidant activity and level, resulting in the development of oxidative stress [27]. Patients with diabetes have substantially greater levels of oxidative stress than the general population with numerous complications and side effects including male reproduction issues due to reduced sperm quality [31]. Previous studies showed that male sub-/infertility has previously been linked to diabetes-induced hyperglycemia dating back to the late seventies using rat model [32–35]. As plasma insulin does not pass through the blood-testis barrier [36], controlling hyperglycemia in patients have been linked to improving testicular oxidative damage [37]. In line with previous studies, the current study evidenced that glycemic control may play a key role in reducing DM-related subfertility or infertility problems by acting on oxidative stress biomarkers.

In males, the main source of androgens, or testosterone, is the Leydig cell. Their physiology enables them to be essential to numerous key physiological processes in males, including as spermatogenesis, the creation of sperm and the regulation of sexual development. Cadmium or hydrogen peroxide treatment of cultured Leydig cell lines resulted in decreased StAR expression [38]. Reversing the decline in testicular StAR mRNA expression seen in adult rats exposed to the heavy metal cadmium provides evidence in favor of ROS-mediated pathways contributing to the arsenic effect [39]. Likewise, in our study, we have seen a significant decrease in *StAR* gene expression resulting from moderate hyperglycemia and increased ROS activity within DM group. As such, EMPA treatment has actually reversed those side effects.

Alongside controlling hyperglycemia either though diet [40], medicinal herbs [41] or drugs such as metformin or empagliflozen [42], the use of antioxidants have been widely advocated to help patients with diabetes avoid problems by reducing oxidative stress, either indirectly through improved glucose management and/or directly by scavenging free radicals or boosting antioxidant defenses [43].

Hyperglycemia and insulin resistance have also been associated altered reproductive hormonal levels, in particular oestradiol and testosterone levels, resulting in decreased sperm quality [44, 45]. In our study, we did not detect any change in hormonal levels and this could possibly be due to the concentration of STZ used was not enough to induce DM but rather moderate hyperglycemia 2) the short length of time, only 8 weeks, was not enough to see the full effect of EMPA and its full curative outcomes [46].

The testes are essential organs in male reproduction being involved in spermatogenesis and testosterone secretion. As such, numerous studies using male animal models revealed in the activity levels of androgen dependent enzymes under hyperglycemia and induced DM altered testicular activities in comparison to control gonads, especially SDH and ACP [47]. They have needed for optimal germ cell proliferation and are linked to spermatogenesis. SDH is an catalyst of oxidation reduction reaction interconverting fructose and sorbitol and because SDH is a marker enzyme that triggers the spermatogenesis process, a decrease in SDH activity in the testis under diabetes indicates that spermatogenesis may be impaired [48]. SDH circulates and is located in the seminiferous tubules and germ cells involved in the energy metabolism of spermatozoa and maturation of spermatogenic cells [49]. In our present study, decreased level of SDH in the STZ-induced hyperglycemia group was noticed and with EMPA treatment on have shown a trend in mediating SDH activities. The enzyme activity results along with histological testicular tissue improvements found in EMPA treated group indicated that the control of hyperglycemia is important in maturation and energy metabolism of spermatogenic cells and spermatozoa.

Additionally, spermatogenesis is highly controlled by cytokines. As such, male infertility has also been linked to inflammatory biomarkers such as IL-6 and TNF-α and -β by disrupting the penile endothelium though increasing ROS testicular tissue levels [50]. Cytokines are secreted by a number of immune cells in the male genital tract, including macrophages, monocytes, and lymphocytes, as well as in response to external antigens and pathogens during chronic inflammation [51], while also having a critical function during the maturation cycles of seminiferous epithelium through their cyclical production from spermatogenic cells [52]. Conversely, the relationship between IL-6, a pro-inflammatory cytokine, and semen quality is still under investigation with most studies reporting a link with altered sperm motility [53]. Our *in vivo* experimental study supports the role of IL-6 as its levels being elevated in STZ-induced hyperglycemia in male wistar rats and EMPA treated groups shown an amended levels of IL-6, in consistency with other studies both in vivo and in vitro [54, 55].

In conclusion, the present study further suggested the protective effects of EMPA and how it has a beneficial role and can effectively attenuate hyperglycemia-induced testicular oxidative damage and inflammatory markers as well as androgen dependent testicular enzymes activity as a protective role against the consequences of hyperglycemia and male sub-/infertility. Future research is highly encouraged towards the design of randomized and controlled clinical trials aimed at evaluating the effects of EMPA on the testicular function [56].

## Supporting information

S1 Data Raw data of the hormonal levels, antioxidant and enzymes activity estimated in Testicular Serum through enzyme-linked immunosorbent assay (ELISA). (XLSX)

S2 Data Raw data of quantitative real-time PCR result of AMPK and StAR gene expression. (XLSX)

## Abbreviations

EMPA: Empagliflozin
STZ: streptozotocin
TBARS: thiobarbituric acid reactive substances
SOD: superoxide dismutase
GPX: glutathione peroxidase
NO: penile nitric oxide
TAC: total anti-oxidant capacity
IL-6: tissue interleukin 6
TGF-β1: transforming growth factor beta 1
ACP: Acid Phosphatase
CK: Creatine Kinase
G6PD: Glucose-6-Phosphate Dehydrogenase
SDH: Sorbitol Dehydrogenase
GnRH: gonadotropin-releasing hormone
FSH: follicle stimulating hormone
LH: luteinizing hormone
DM: Diabetes mellitus
AMPK: AMP-activated protein kinase
StAR: Steroidogenic acute regulatory protein
